# Regulation of secondary growth by poplar *BLADE-ON-PETIOLE* genes in Arabidopsis

**DOI:** 10.3389/fpls.2023.1244583

**Published:** 2023-11-14

**Authors:** Sibei Li, Bhaswati Devi, Gamalat Allam, Armaan Bhullar, Jhadeswar Murmu, Eryang Li, Shelley R. Hepworth

**Affiliations:** Department of Biology, Carleton University, Ottawa, ON, Canada

**Keywords:** BLADE-ON-PETIOLE, TGA bZIP, *Populus trichocarpa*, secondary growth, lignin

## Abstract

*BLADE-ON-PETIOLE* (*BOP*) genes are essential regulators of vegetative and reproductive development in land plants. First characterized in *Arabidopsis thaliana* (Arabidopsis), members of this clade function as transcriptional co-activators by recruiting TGACG-motif binding (TGA) basic leucine zipper (bZIP) transcription factors. Highly expressed at organ boundaries, these genes are also expressed in vascular tissue and contribute to lignin biosynthesis during secondary growth. How these genes function in trees, which undergo extensive secondary growth to produce wood, remains unclear. Here, we investigate the functional conservation of *BOP* orthologs in *Populus trichocarpa* (poplar), a widely-used model for tree development. Within the poplar genome, we identified two *BOP*-like genes, *PtrBPL1* and *PtrBPL2*, with abundant transcripts in stems. To assess their functions, we used heterologous assays in Arabidopsis plants. The promoters of *PtrBPL1* and *PtrBPL2*, fused with a β-glucuronidase (GUS) reporter gene showed activity at organ boundaries and in secondary xylem and phloem. When introduced into Arabidopsis plants, *PtrBPL1* and *PtrBPL2* complemented leaf and flower patterning defects in *bop1 bop2* mutants. Notably, Arabidopsis plants overexpressing *PtrBPL1* and *PtrBPL2* showed defects in stem elongation and the lignification of secondary tissues in the hypocotyl and stem. Finally, PtrBPL1 and PtrBPL2 formed complexes with TGA bZIP proteins in yeast. Collectively, our findings suggest that PtrBPL1 and PtrBPL2 are orthologs of Arabidopsis BOP1 and BOP2, potentially contributing to secondary growth regulation in poplar trees. This work provides a foundation for functional studies in trees.

## Introduction

*Populus trichocarpa* is a deciduous tree species that is commonly known as black cottonwood or western balsam poplar. A long-lived, mostly diploid species that is native to western North America, it is widely distributed in temperate and cold temperate regions (Cooke and Rood, 2007). *P. trichocarpa* and companion species in the genus *Populus* (poplar) are valuable model organisms for tree research (Taylor, 2002; Jansson and Douglas, 2007). The genus is closely related to the model plant *Arabidopsis thaliana* (Arabidopsis) and many poplar gene functions are conserved. An available variety of molecular tools, such as genetic transformation methods, CRISPR-based gene editing systems, and a growing number of sequenced genomes, make poplar species ideal for molecular studies (Jansson and Douglas, 2007; Bryant et al., 2020).

BLADE-ON-PETIOLE (BOP) co-transcriptional regulators, first described in Arabidopsis, belong to a family in land plants that have a BTB/POZ (Broad-complex, Tramtrack, and Bric-a-brac/POX virus and zinc finger) domain and ankyrin repeats for interaction with other proteins (Khan et al., 2014; Backer et al., 2019). Family members are classified into two phylogenetic subclades (Khan et al., 2014; Backer et al., 2019). The first subclade contains NPR1-type proteins involved in plant defense whereas BOP-type proteins primarily regulate plant development (Khan et al., 2014; Backer et al., 2019). Proteins from both subclades lack a DNA binding domain and interact TGACG-motif binding (TGA) basic leucine zipper (bZIP) transcription factors for the co-activation or co-repression of target genes. BTB-ankyrin proteins from both subclades can also function as E3 ubiquitin ligase adaptors involved in regulating protein abundance (Fu et al., 2012; Zhang et al., 2017; Chahtane et al., 2018; He et al., 2020). In monocots and dicots, BOPs contribute to a surprisingly large number of developmental processes, including lignin deposition as a defense (Zhang et al., 2019) and during secondary growth (Khan et al., 2012b; Woerlen et al., 2017; Shen et al., 2021; Liu et al., 2022).

In Arabidopsis, *BOP1* and *BOP2* genes are strongly expressed at organ boundaries, zones that connect organs to the plant body (Žádníková and Simon, 2014; Hepworth and Pautot, 2015). These areas regulate growth at the base of organs and produce axillary meristems for the development of lateral branches, flowers, and appendages such as stipules or nectaries. Boundaries are also sites where organs separate by abscission or dehiscence to release foliage, fruits, or seeds (Hepworth and Pautot, 2015). Phenotypic defects in *bop1 bop2* double mutants are concentrated at lateral organ boundaries resulting in elongated leafy petioles, five-petalled flowers with a subtending bract, and defects in abscission (Hepworth et al., 2005; Norberg et al., 2005; McKim et al., 2008). In monocots and dicots, BOP1 and BOP2 paralogs participate in many of the same developmental processes (Wu et al., 2012; Tavakol et al., 2015; Couzigou et al., 2016; Jost et al., 2016; Xu et al., 2016; Toriba et al., 2019; Magne et al., 2020; Liu et al., 2022).

In Arabidopsis, *BOP1* and *BOP2* genes are also expressed in vascular tissues (Hepworth et al., 2005; Khan et al., 2012a). The overexpression of either gene inhibits stem elongation and disrupts secondary growth in stems and the root-hypocotyl (Khan et al., 2012a; Woerlen et al., 2017). In the inflorescence stem, lignified phloem and interfascicular fibers are completed earlier and the central pith becomes lignified in severe lines (Khan et al., 2012a). Secondary growth in the root-hypocotyl is also disrupted, with loss of xylem fiber differentiation (Liebsch et al., 2014; Woerlen et al., 2017). Unlike trees, secondary growth in the majority of the Arabidopsis stem is limited to the secondary thickening of xylem and phloem cell walls and the lignification of interfascicular fibers spaced between the primary vascular bundles. These fibers complete the vascular ring and provide mechanical support (Rogers and Campbell, 2004; Ehlting et al., 2005).

In woody dicot plants, cambium activity initiates in the primary vascular bundles (fascicular cambium) and spreads out to the interfascicular regions where differentiated cells regain the ability to divide (interfascicular cambium). In Arabidopsis, continuous vascular cambium only forms in the root-hypocotyl and at the base of the primary inflorescence 1-2 mm above the rosette (Sehr et al., 2010; Sanchez et al., 2012). The end result is a tube-like sheath of meristematic activity. Daughter cells on the outer rim of the cambium produce secondary phloem (inner bark) whereas daughter cells on the inside make secondary xylem (wood). In forest trees, this activity results in the radial thickening of stems and supports the development of large body architectures (Groover et al., 2006; Sehr et al., 2010; Sanchez et al., 2012).

*P. trichocarpa* and its relatives have emerged as an excellent platform for the study of trees (Jansson and Douglas, 2007; Bryant et al., 2020). The industrial impact of poplar is significant as hybrid poplars are among the fastest growing temperate forest trees in the world, ready for harvesting at 10 to 20 years. Trees can be grown on forest lands or marginal crop lands. Plantations, totalling some 9.6 million hectares worldwide, contribute to the storage of atmospheric CO_2_ as biomass, which is harvested for the production of wood chips, plywood, biofuels, and other materials. Lignin and wood engineering in forest trees can benefit from a detailed understanding of the molecular mechanisms controlling tree architecture and wood development (Sannigrahi et al., 2010; Chanoca et al., 2019).

Here, we investigate the function of two *BOP*-like genes in *P. trichocarpa*. Highly expressed in poplar xylem and phloem, we provide evidence that these genes when heterologously expressed in Arabidopsis can function at organ boundaries and regulate lignin deposition in tissues undergoing secondary growth. We also provide evidence of complex formation with TGA bZIP proteins indicating that gene functional networks might be conserved in poplar trees. The potential evolutionary implications of these findings are discussed.

## Materials and methods

### Plant material and growth conditions

The *P. trichocarpa* female clone “Nisqually-1” was used (Song et al., 2006). Cuttings from a tree grown on the University of British Columbia campus (a gift of Sean Mansfield) were rooted in soil and grown under natural lighting in a greenhouse. The *Arabidopsis thaliana* Columbia (Col-0) accession was used as the wild type. The double mutant *bop1 bop2* (Hepworth et al., 2005), activation-tagged overexpression line *bop1-6D* and transgenic line *35S:BOP2* (Norberg et al., 2005) were previously described. Surface-sterilized seeds were sown on minimal media agar plates (Haughn and Somerville, 1986). Ten-day-old seedlings were planted in sterilized soil (Promix BX, Premier Tech, Quebec) supplemented with 20-20-20 fertilizer (Plant Product Co. Ltd, Brampton, Ontario). The plants were grown to maturity in chambers at 21°C under long days (8 h dark/16 h light, intensity 100 µmol m^-2^ s^-1^).

### *PtrBPL1* and *PtrBPL2* complementation of Arabidopsis *bop1 bop2* mutant

*PtrBPL1* and *PtrBPL2* genes were expressed under the control of a *BOP1* promoter in Arabidopsis *bop1 bop2* mutant plants. This promoter has been previously used for complementation studies (Khan et al., 2015). The cDNA sequences of *PtrBPL1* (Potri.016G040500) and *PtrBPL2* (Potri.006G043400) were amplified by polymerase chain reaction (PCR) using cDNA from mixed poplar tissue as the template and high-fidelity iProof polymerase (Biorad, Hercules, CA). The primers annealed to the 5’ and 3’ untranslated regions of each gene to ensure that amplification was specific. The resulting products were cloned into pCR™-BluntII-TOPO™ (Invitrogen, ThermoFisher Scientific, Waltham, MA) and verified by DNA sequencing (Eurofins Genomics, Louisville, KY). Verified clones were used as template to amplify the coding regions of *PtrBPL1* and *PtrBPL2* using PtrBPL-XbaI-F and PtrBPL-Sac1-R as the primers. The resulting products were cloned into the corresponding sites of binary vector pBAR (a gift from the Dangl lab, University of North Carolina) downstream of the *AtBOP1* promoter (McKim et al., 2008). The resulting constructs named *BOP1p:PtrBPL1* and *BOP1p:PtrBPL2* were introduced into *Agrobacterium tumefaciens* strain C58C1 pGV101 pMP90 (Koncz and Schell, 1986). Arabidopsis *bop1 bop2* plants were transformed by floral dipping (Clough and Bent, 1998). Glufosinate-resistant primary transformant (T1) plants were selected on soil using the herbicide Finale (Bayer Environmental Sciences, Sacramento, CA). Complementation of *bop1 bop2* leaf, flower, and abscission defects was scored in the T1 generation. The progeny of ten independent T1 lines showing strong complementation were genotyped to confirm that they were *bop1 bop2* double mutants. Three to five independent transgenic lines were selected for further analysis. Primers used for cloning are listed in **Supplementary Table S1**.

### *PtrBPL1* and *PtrBPL2* promoter GUS reporter constructs

To determine the expression pattern of *PtrBPL1* and *PtrBPL2* genes, the promoters were PCR-amplified from genomic DNA template extracted from young poplar leaves using a Genomic DNA Mini Kit (Plant) (FroggaBio Inc., Concord, Ontario). A 3938-bp *PtrBPL1* promoter (nucleotides -3867 to +112) was assembled using an overlap PCR approach because the full-length sequence was difficult to amplify. A 2.9-kb *PtrBPL1* promoter fragment (nucleotides -3867 to -2876) was PCR-amplified using ptBPL1-pro-F10 and ptBPL1-pro-R1 as the primers. A 1.1-kb *PtrBPL1* promoter fragment (-2807 to +112) was PCR amplified using BPL1-pro-F8 and BPL1-May14-R as the primers. These two *PtrBPL1* overlapping promoter fragments were gel purified, mixed, and used as template to amplify a full-length product using PtBPL1-pro-F10 and BPL1-May14-R as the primer pair. The resulting *PtrBPL1* promoter (including 112-bp of *PtrBPL1* coding sequence) was column-purified and cloned into a Zero Blunt™ TOPO™ vector (Invitrogen, ThermoFisher Scientific). DNA sequencing identified a 42-bp sequence deletion (nucleotides -3757 to -3715) compared to publicly available database sequence. To create the reporter gene, the *PtrBPL1* promoter was amplified from the above plasmid using BPL1-pro-BamH1-F and BPL1-pro-Nco1-R as the primer pair. The resulting product was column-purified, digested with *Bam*H1 and *Nco*1 restriction enzymes and ligated into the corresponding sites of the pGreen-based pTGA9_pro_:GUS plasmid (Hellens et al., 2000; Murmu et al., 2010). This ligation placed the *PtrBPL1* promoter upstream of a GUS reporter gene as a translational fusion. Similarly, a 4092-bp *PtrBPL2* promoter sequence (nucleotide -4059 to +33) was PCR-amplified using BPL2-4kb-F and BPL2-Promo-R as the primers and cloned into the Zero Blunt™ TOPO™ vector (Invitrogen, ThermoFisher Scientific). The *PtrBPL2* promoter insert was then amplified using BPL2-pro-BamH1-F and BPL2-pro-Nco1-R as the primers, digested with *Bam*HI and *Nco*I restriction enzymes, and cloned into the corresponding sites of pTGA9_pro_:GUS (Murmu et al., 2010). DNA fragments destined for cloning were amplified using iProof high-fidelity polymerase (Biorad, Hercules, CA). All clones were verified by DNA sequencing (Eurofins Genomics). The resulting GUS reporter constructs were co-transformed with pSOUP into *Agrobacterium tumefaciens* strain C58C1 pGV101 pMP90 (Koncz and Schell, 1986; Hellens et al., 2000). Wild-type plants were transformed by floral dipping (Clough and Bent, 1998). Glufosinate-resistant primary transformant (T1) plants were selected on soil using the herbicide Finale (Bayer Environmental Sciences). Multiple independent transgenic lines per construct were evaluated for reporter GUS activity. Three independent transgenic lines were selected for further analysis. Primers used for cloning are listed in **Supplementary Table S1**.

### Constructs for overexpression of *PtrBPL1* and *PtrBPL2*

The coding sequences of *PtrBPL1* and *PtrBPL2* were PCR-amplified from cloned cDNA template using iProof as the polymerase (Biorad) and Pt6s04010CDS-F and Pt6s04010CDS-R primer set, and Pt6s04190CDS-F and Pt6s04190CDS-R primer set, respectively. The resulting products were placed into the Gateway entry vector pCR™8/GW/TOPO™ (Invitrogen, ThermoFisher Scientific) and moved into the pSM3 binary vector (Unda et al., 2017) downstream of a double 35S CaMV promoter (D35S) using Gateway™ LR Clonase™ (Invitrogen, ThermoFisher Scientific). Wild-type plants were transformed as described above. Hygromycin-resistant primary transformants were selected on agar plates. Phenotypes were scored in the T1 generation. Three to five independent transformants were selected for further analysis. Primers used for cloning are listed in **Supplementary Table S1**.

### Reverse transcription-quantitative PCR

Total RNA was isolated from dissected poplar tissues (a gift of the Carl Douglas lab). 2 µg of RNA was used for cDNA synthesis using Superscript III reverse transcriptase (Invitrogen, ThermoFisher Scientific). PCR reactions in triplicate containing 2 µl of 10-fold diluted cDNA, gene-specific primers (**Supplementary Table S1**) and Power SYBR™ Green Master mix (Invitrogen, ThermoFisher Scientific) were carried out using a StepOnePlus thermocycler (Applied Biosystems, ThermoFisher Scientific). Relative transcript levels were calculated according to Pfaffl (2001). Values were normalized to the poplar elongation factor reference gene *C672* (Wang et al., 2014) and then to young leaf. Data are the average of four measurements for each of two biological replicates. Error bars show standard deviation.

### Localization of lignin deposition

Tissues were analyzed for lignin deposition as previously described (Khan et al., 2012b; Woerlen et al., 2017). Arabidopsis stems were harvested from five-week-old plants. A razor blade was used to hand-cut sections from the base of the primary inflorescence about 1 cm above the rosette leaves (Khan et al., 2012b). Hypocotyls were harvested from seven-week-old plants. A razor blade was used to hand-cut sections about 1.5 mm below the rosette leaves. Samples were placed in 2% phloroglucinol dissolved in 95% ethanol for five minutes. Then, five drops of concentrated hydrochloric acid were added. Two minutes were allowed for color development. Immediately, samples were transferred onto a glass slide and a cover slip was added. Images were collected using a Discovery V20 stereomicroscope (Carl Zeiss Canada, North York, Ontario).

### Localization of GUS activity

Tissues were analyzed for β-glucuronidase (GUS) activity as previously described (Woerlen et al., 2017) with minor changes. The staining solution contained 4 mM KFe(CN) and 2 mM of 5-bromo-4-chloro-3-indoxyl-β-D-glucuronide (X-Gluc). The samples were incubated at 37°C for 3 to 24 hours until a localized blue precipitate was visible. After clearing in 70% ethanol, the samples were imaged using a Discovery V20 stereomicroscope (Carl Zeiss). For sections, the stained tissue was embedded in Paraplast Plus^®^ (Sigma-Aldrich, St. Louis, MO) and processed using *tert*-butanol instead of xylenes (Woerlen et al., 2017). Tissue sections (20 µm) were fixed onto glass slides and dewaxed with *tert*-butanol. The samples were imaged using an Axio Imager M2 compound microscope (Carl Zeiss).

### Yeast two-hybrid assay

Protein-protein interactions were assayed using a Matchmaker™ GAL4-based yeast two-hybrid system (Clontech, Takara Bio USA Inc, San Jose, CA) and Gateway-compatible pGBKT7-DEST (bait) and pGADT7-DEST (prey) plasmids modified from pGBKT7 and pGADT7-Res vectors, respectively (Lu et al., 2009). PtrBPL1 and PtrBPL2 proteins fused to the DNA-binding domain of yeast GAL4 were used as bait. AtTGA1, AtTGA4, AtTGA3, AtTGA7 and AtTGA8/PAN proteins fused to the transcriptional activation domain of yeast GAL4 were used as prey. To prepare Gateway entry vectors, the full-length coding sequences of bait and prey genes were cloned into pCR™8/GW/TOPO™TA (Invitrogen, ThermoFisher Scientific). All entry vector inserts were sequenced to confirm authenticity. To make the final plasmids, recombination reactions were performed using Gateway™ LR Clonase™ II enzyme mix according to the manufacturer’s instructions (Invitrogen, ThermoFisher Scientific). Bait and prey plasmid were co-transformed into yeast AH109 strain (Gietz and Schiestl, 2007). Transformed yeast colonies were identified by selection on synthetic complete (SC) media plates lacking Leu and Trp (SC/-Leu/-Trp). Dilution series were spotted onto SC/-Leu/-Trp medium or SC/-Leu-Trp-His medium plus 10 mM 3-amino-1,2,4-triazole (3-AT; Sigma-Aldrich) for assessment of His reporter gene activity. A 3-AT concentration (10 mM) sufficient to distinguish positive growth from background was determined empirically.

### Bioinformatics

*P. trichocarpa* homologs of Arabidopsis BTB-ankyrin and TGA bZIP proteins were identified using the plant homologs tool at The Arabidopsis Information Resource (TAIR) (www.arabidopsis.org). The corresponding *P. trichocarpa* protein sequences were retrieved from Phytozome (Goodstein et al., 2012; phytozome-next.jgi.doe.gov). MEGA version 11 was used for the alignment of protein sequences by MUSCLE (Edgar, 2004) using the default parameters (www.megasoftware.net). Maximum Likelihood trees were constructed based on 100 bootstrap replicates using the Jones-Taylor-Thornton Model. The BOXSHADE alignment and sequence logos were prepared using Geneious Prime 2022.1 (www.geneioius.com). The percent similarity of protein pairs was calculated using the Expasy SIM tool (www.expasy.org/sim).

### Promoter analysis

The 500-bp promoter sequences upstream of the translational start sites of *AtBOP1*, *AtBOP2*, *PtrBPL1*, and *PtrBPL2* genes were retrieved from their genome assemblies found at TAIR (https://www.arabidopsis.org/) or Phytozome (https://phytozome-next.jgi.doe.gov/). Scanning for Transcription Factor Binding Sites (TFBS) was carried out using PlantPAN 3.0 (http://plantpan.itps.ncku.edu.tw/index.html). Selected TFBS were aligned to statistically enriched 6-mers identified by the motif finder tool at TAIR. Binding site locations were visualized by using TBtools v1.123 (Chen et al., 2020).

## Results

### Identification of BTB-ankyrin and TGA bZIP gene families in poplar

**Figure 1:**
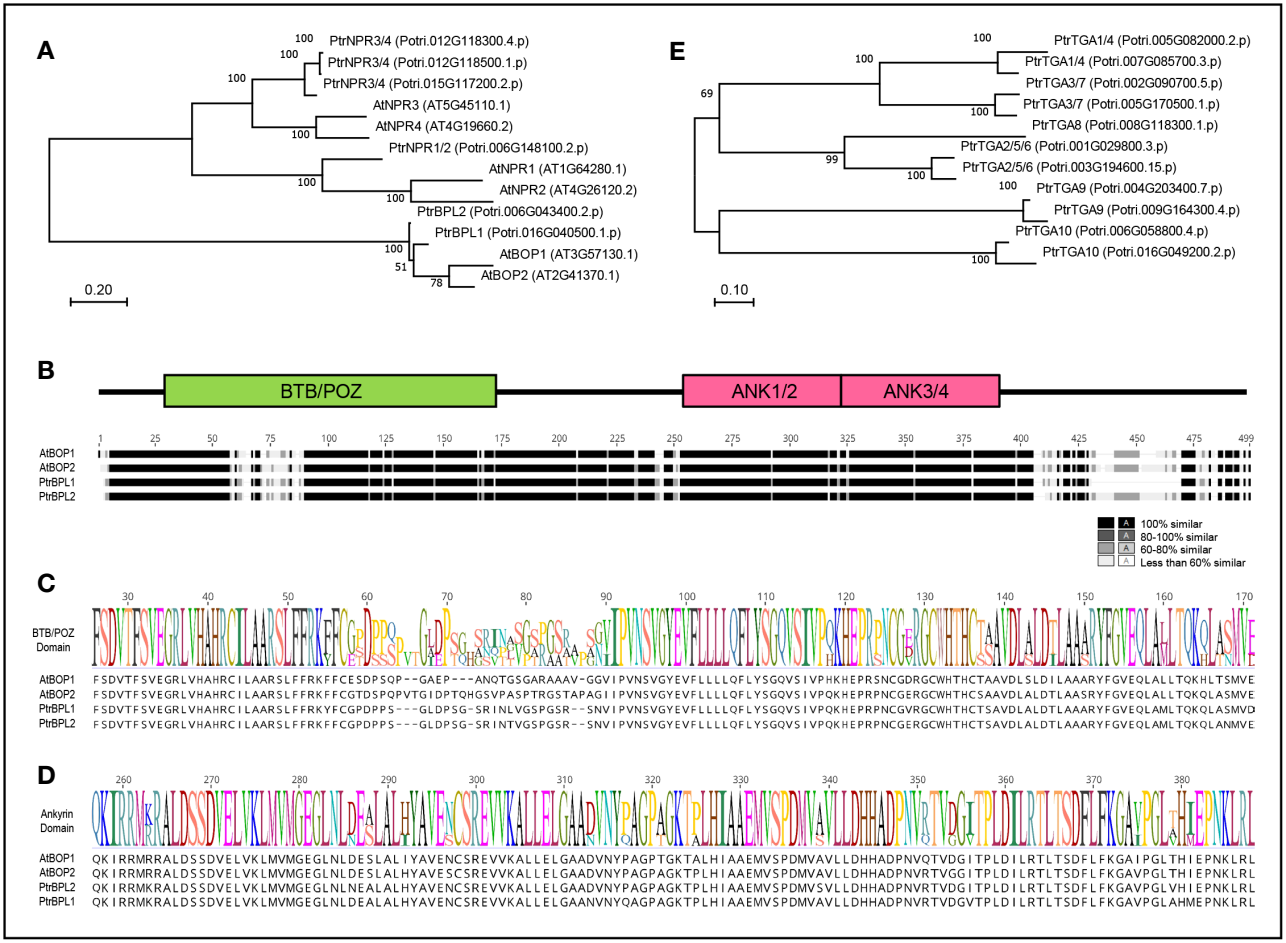
Proteins encoded by BTB-ankyrin and TGA bZIP genes from Arabidopsis and poplar. **(A)** Maximum Likelihood tree showing phylogenetic relationships between Arabidopsis (At) and poplar (Ptr) BTB-ankyrin proteins. **(B)** Multiple sequence alignment of AtBOP and PtrBPL proteins with corresponding domain map showing the relative position of conserved BTB/POZ and ankyrin (ANK) domains. Segments of highest similarity or identity are colored darkest and segments of lowest identity or similarity are colored lightest. **(C)** BTB/POZ domain sequence logo. **(D)** Ankyrin domain sequence logo. Protein sequences with listed identification numbers were retrieved from TAIR (www.arabidopsis.org) or Phytozome (phytozome-next.jgi.doe.gov). **(E)** Maximum Likelihood tree showing phylogenetic relationships between PtrTGA proteins.

In the *P. trichocarpa* (poplar) genome, six BTB-ankyrin proteins were identified. The phylogenetic relationship Arabidopsis and poplar BTB-ankyrin proteins was investigated (**Figure 1A**). This analysis revealed one NPR1 protein (Potri.006G148100), three NPR3/4 proteins (Potri.012G118300; Potri.012G118500; Potri.015G117200) and two poplar BOP-like proteins designated as PtrBPL1 (Potri.016G040500) and PtrBPL2 (Potri.006G043400), respectively. Multiple sequence alignment showed that PtrBPL1 and PtrBPL2 are 94.3% similar to each other at the amino acid level and 80.2% versus 75.6% similar to AtBOP1, respectively. The similarity is broadly distributed across the length of the proteins, not just within the BTB/POZ and ankyrin repeat domain (**Figures 1B–D**; **Supplementary Figure S1**). Less conserved regions are situated within the BTB/POZ domain and near the C-terminus. For example, PtrBPL1 contains a deletion in the C-terminus compared to AtBOP1/2 and PtrBPL2 proteins (**Figure 1B**; **Supplementary Figure S1**). In parallel, we investigated the phylogenetic relationship of poplar TGA bZIP proteins as potential functional partners. Representatives from all five TGA clades found in Arabidopsis are present (**Figure 1E**; **Supplementary Figure S2**). Given these overall similarities, we anticipated that PtrBPL1 and PtrBPL2 might have conserved activities similar to AtBOP1 and AtBOP2.

### *PtrBPL1* and *PtrBPL2* expression pattern

**Figure 2:**
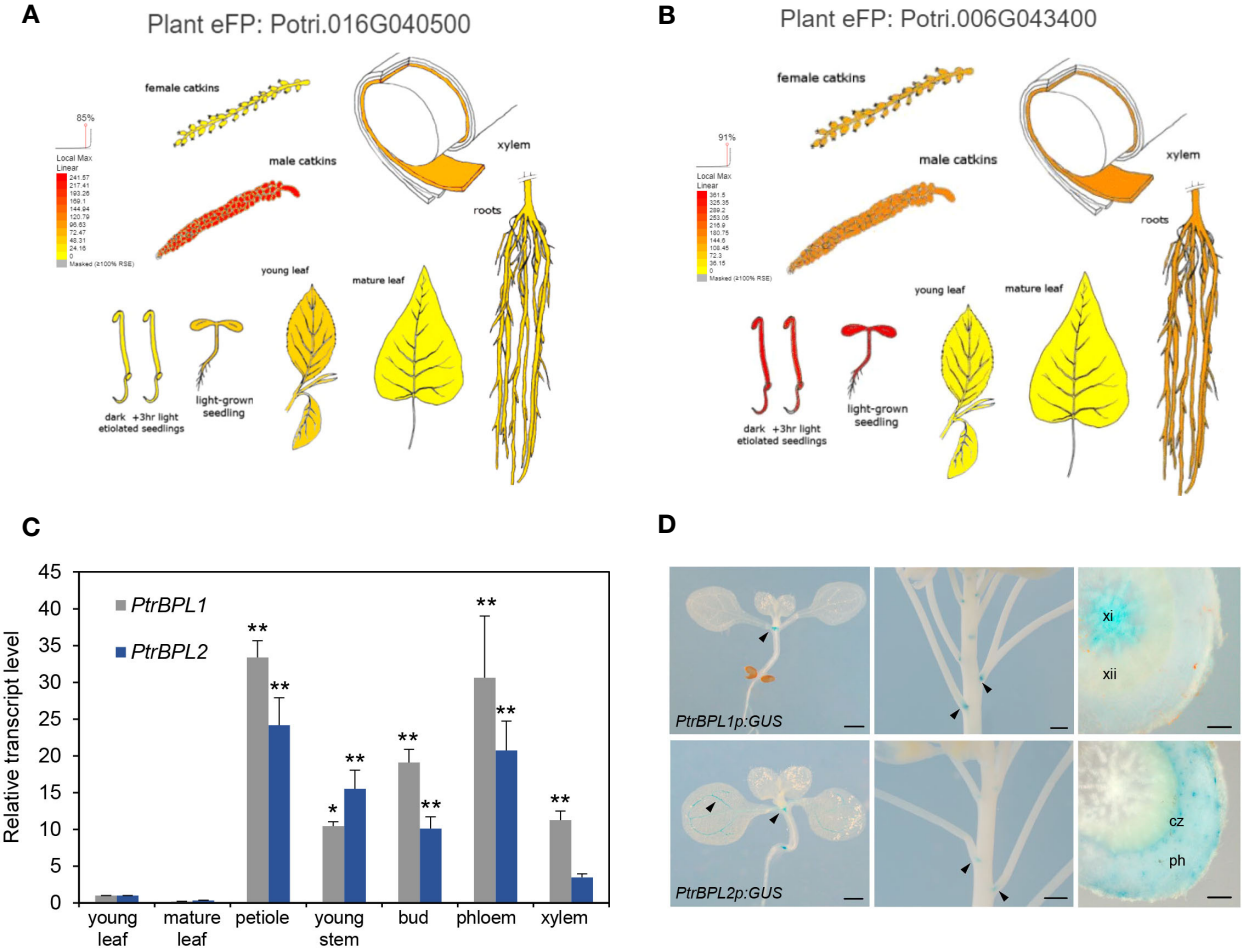
Expression of *PtrBPL1* and *PtrBPL2* in poplar and Arabidopsis. **(A, B)** Transcriptome data for *P. trichocarpa* compiled by the Bio-Analytic Resource for Plant Biology (www.bar.utoronto.ca). Red, high expression; orange, medium expression; yellow, lower expression. Plant eFP viewer output for **(A)** *PtrBPL1* and **(B)** *PtrBPL2*. **(C)** The relative abundance of *PtrBPL1* and *PtrBPL2* transcript was independently measured using RT-qPCR in dissected poplar tissues. Data are the average ± SD of four measurements performed on each of two biological replicates. Values were normalized to young leaf. Asterisks indicate significant differences between tissues compared with young leaf (ANOVA with Tukey’s test, **P<0.01, *P<0.05). **(D)** Expression patterns of *PtrBPL1p:GUS* and *PtrBPL2p:GUS* in Arabidopsis seedlings, inflorescences, and sectioned hypocotyls. Arrows denote expression at organ boundaries. xi, secondary xylem I; xii, secondary xylem II; ph, secondary phloem; cz, cambium zone. Scale bars, 500 µm except 100 µm for hypocotyl sections.

We next obtained expression data for *PtrPBL1* and *PtrBPL2* from the Bio-Analytic Resource for Plant Biology (BAR, www.utoronto.ca/bar). These data showed the wide expression of both genes in poplar tissues including seedlings, young leaves, catkins, roots, and xylem (**Figures 2A, B**). We then used RT-qPCR to monitor transcript abundance in selected tissues. These data showed that *PtrBPL1* and *PtrBPL2* transcripts are enriched in the petiole region of leaves compared to the blade. Significant expression was also observed in xylem and phloem tissues of young poplar stems (**Figure 2C**).

We next investigated the spatial and temporal expression of *PtrBPL1* and *PtrBPL2* genes, by monitoring the expression of *PtrBPL1p:GUS* and *PtrBPL2p:GUS* reporter genes in Arabidopsis plants (**Figure 2D**). In seedlings, both genes were expressed in boundaries at the base of the cotyledons. During flowering, both genes were expressed at nodes in the stem where a boundary forms at the base of the flower pedicel. GUS expression was evident in floral organ boundaries and abscission zones at the base of young siliques for *PtrBPL1* but not *PtrBPL2* (**Supplementary Figure S3**). The vasculature of cotyledons, leaves, and the hypocotyl also showed differential expression. *PtrBPL2* but not *PtrBPL1* was expressed in veins of leaves (**Figure 2D**; **Supplementary Figure S3**). At the seedling stage, both genes were expressed at the root tip and at the base of lateral roots (**Supplementary Figure S3**). In the hypocotyl, *PtrBPL1* was strongly expressed in early secondary xylem (xylem I) whereas *PtrBPL2* was expressed at the outer edge of the cambial zone and in secondary phloem (**Figure 2D**; **Supplementary Figure S3**). Neither *AtBOP1* nor *AtBOP2* are expressed in secondary xylem (Liebsch et al., 2014; Woerlen et al., 2017) indicating a possible difference in gene regulation between the two species. To investigate this difference, a statistical motif analysis was carried out using the promoter regions (500 base pairs upstream of the start codon) of all four genes. This analysis showed an enrichment of MYB-related and NAC transcription factor binding sites in the promoters of *PtrBPL1* and *PtrBPL2* compared to *AtBOP1* and *AtBOP2* (**Supplementary Figure S4**) implicating these factors in the differential regulation of poplar orthologs.

### *PtrPBL1* and *PtrBPL2* can complement *bop1 bop2* leaf and flower patterning defects

**Figure 3:**
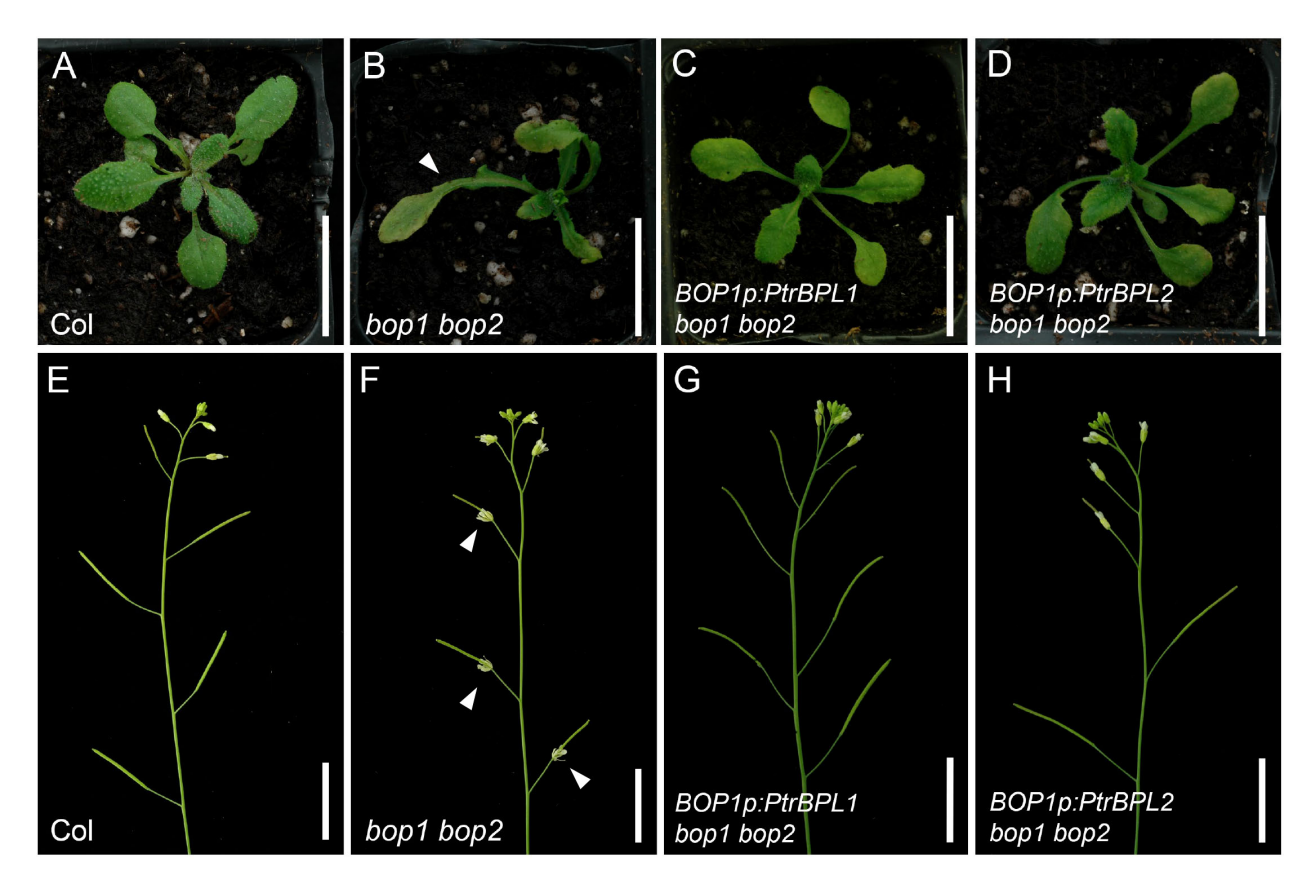
Complementation of Arabidopsis *bop1 bop2* leaf and flower defects with *PtrBPL1* and *PtrBPL2*. *PtrBPL1* and *PtrBPL2* coding regions were expressed under the control of the *AtBOP1* promoter in *bop1 bop2* plants. Representative plants are shown in the T2 generation. See **Supplementary Table S2** for quantitative analysis of complementation for *bop1 bop2* defects in leaf morphology, floral patterning, and abscission. **(A, B)** Wild-type plant showing **(A)** smooth leaf petioles and **(B)** inflorescence with floral organ abscission. **(C, D)** A *bop1 bop2* mutant showing **(C)** leaves with a blade-on-petiole phenotype (arrow) and **(D)** inflorescence with asymmetric flowers and loss of floral organ abscission (arrows). **(E, F)** A *BOP1p:PtrBPL1 bop1 bop2* plant showing **(E)** smooth leaf petioles and **(F)** inflorescence with floral organ abscission restored. **(G, H)** A *BOP1p:PtrBPL2 bop1 bop2* plant showing **(G)** smooth leaf petioles and **(H)** inflorescence with floral organ abscission restored. Scale bars, 1.5 cm.

To investigate to what extent PtrBPL1 and PtrBPL2 can substitute for AtBOP1 and AtBOP2, we expressed *PtrBPL1* and *PtrBPL2* under the control of an *AtBOP1* promoter in *bop1 bop2* mutants and tested for complementation. In total, we obtained 203 primary (T1) transformants for *BOP1p:PtrBPL1* and 212 primary transformants for *BOP1p:PtrBPL2* (**Supplementary Table S2**). Characteristic defects in *bop1 bop2* mutants include leafy petioles, loss of floral organ abscission, and flowers with a bract and extra petals on the abaxial side (Hepworth et al., 2005). Strong or medium complementation of the leaf phenotype was observed in 54% and 52% of *PtrBPL1* and *PtrBPL2* transformants, respectively (**Figure 3**; **Supplementary Table S2**). Floral patterning defects were significantly restored in 65% and 76% of *PtrBPL1* and *PtrBPL2* transformants scored, respectively (**Supplementary Table S2**). Floral organ abscission was significantly restored in 76% and 62% of *PtrBPL1* and *PtrBPL2* transformants scored, respectively (**Figure 3**; **Supplementary Table S2**). These data confirm that heterologous expression of *PtrBPL1* and *PtrBPL2* can complement *bop1 bop2* mutant phenotypes.

### Overexpression of *PtrBPL1* and *PtrBPL2* alters secondary growth

Plants that overexpress *AtBOP1* or *AtBOP2* are short and bushy, with a wider pattern of secondary lignin deposition in stems (Khan et al., 2012b). Unlike trees, a continuous vascular cambium does not form except at the base of the stem. Instead, secondary growth is evident as the differentiation of abundant lignified interfascicular fibers (Ehlting et al., 2005).

**Figure 4:**
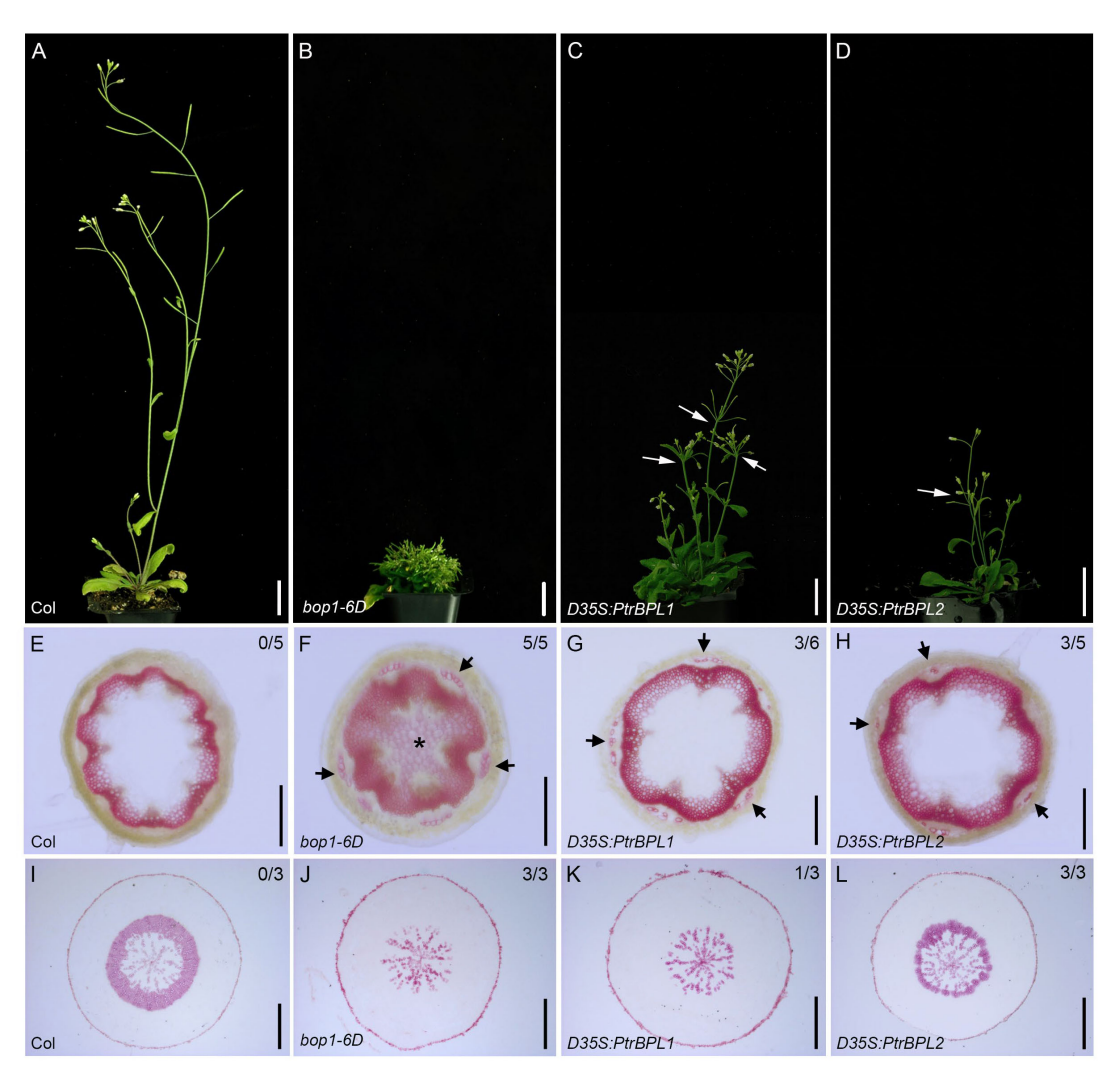
Overexpression of *PtrBPL1* and *PtrBPL2* in Arabidopsis plants. *PtrBPL1* and *PtrBPL2* coding regions were expressed under the control of a double 35S (*D35S*) cauliflower mosaic virus promoter in wild-type Arabidopsis plants. **(A-D)** Representative flowering plants are shown in the T1 generation. See **Supplementary Table S3** for quantitative analysis. **(A)** Wild type plant, showing strong apical dominance and elongated internodes. **(B)** *bop1-6D* plant, showing a bushy, dwarf stature. **(C)** *D35S:PtrBPL1* plant, showing a bushy, semi-dwarf stature. Arrows, clustered flowers and siliques. **(D)** *D35S:PtrBPL2* plant, showing a bushy, semi-dwarf stature. Arrows, clustered flowers and siliques. **(E-H)** Transverse sections from the base of fully elongated stems were stained phloroglucinol-HCl to reveal lignin (pink). Top left, number of independent transgenic lines showing abnormal lignin deposition. Representative sections are shown for: **(E)** wild-type stem, showing a continuous vascular ring. **(F)** *bop1-6D* stem, showing a thicker vascular ring, lignified pith (asterisk) and lignified phloem fibers (arrows) **(G)** *D35S:PtrBPL1* stem, showing a thick vascular ring and lignified phloem fibers (arrows). **(H)** *D35S:PtrBPL2* stem, showing a thick vascular ring and lignified phloem fibers (arrows). **(I-L)** Transverse sections from the middle of the hypocotyl (1.5 mm below the rosette leaves) were stained phloroglucinol-HCl to reveal lignin (pink). Top left, number of independent transgenic lines showing abnormal lignin deposition. Representative sections are shown for: **(I)** Wild-type hypocotyl, showing a thick ring of xylem II. **(J)** *bop1-6D* hypocotyl, showing a lack of xylem II fibers and vessels. **(K)** *D35S:PtrBPL1* hypocotyl, showing a lack of xylem II fibers and vessels. **(L)** *D35S:PtrBPL2* hypocotyl, showing a xylem II ring of reduced thickness, compared to the wild type. Scale bars: **(A-D)** 1.5 cm; **(E-H)** 0.25 mm; **(I-L)** 0.5 mm.

To test the effect of *PtrBPL1* and *PtrBPL2* on stem development, Arabidopsis plants were transformed with a construct driving *PtrBPL1* and *PtrBPL2* expression from a strong, constitutive double cauliflower mosaic virus 35S promoter (D35S). Flowering *D35S:PtrBPL1* and *D35S:PtrBPL2* plants showed a loss of apical dominance and reduced stature in comparison to wild-type plants (**Figure 4**; **Supplementary Table S3**). The stems of 5-6 representative *D3SS : PtrBPL1* and *D35S:PtrBPL2* plants were hand-sectioned and stained with phloroglucinol to test for changes in lignin deposition. Several plants in this population showed an expanded pattern of lignin deposition in stems.

In the root-hypocotyl where a continuous vascular cambium forms, two phases of secondary growth take place (Chaffey et al., 2002; Nieminen et al., 2015). During the first phase, the xylem I is composed of lignified vessel cells in a matrix of non-lignified parenchyma. Flowering triggers the second phase, in which xylem II forms as a thick ring composed of lignified fibers interspersed with lignified vessels (Chaffey et al., 2002; Nieminen et al., 2015). Arabidopsis plants that overexpress *AtBOP1* and *AtBOP2* lack xylem II features in the upper part of the root (Woerlen et al., 2017) and hypocotyl (Liebsch et al., 2014).

Analysis of secondary growth in the hypocotyl of *D35S:PtrBPL1* and *D35S:PtrBPL2* lines showed at least one *D35S:PtrBPL1* line (n=3) missing xylem II fibers and vessels similar to *bop1-6D* and *35S:BOP2* control plants (**Figure 4**; **Supplementary Figure S5**). The xylem II ring in all *D35S:PtrBPL2* lines (n=3) was reduced in thickness compared to wild-type control plants. These data provide further evidence that *PtrBPL1* and *PtrBPL2* can functionally substitute in Arabidopsis plants.

### PtrBPL1 and PtrBPL2 interact with TGA bZIP factors

**Figure 5:**
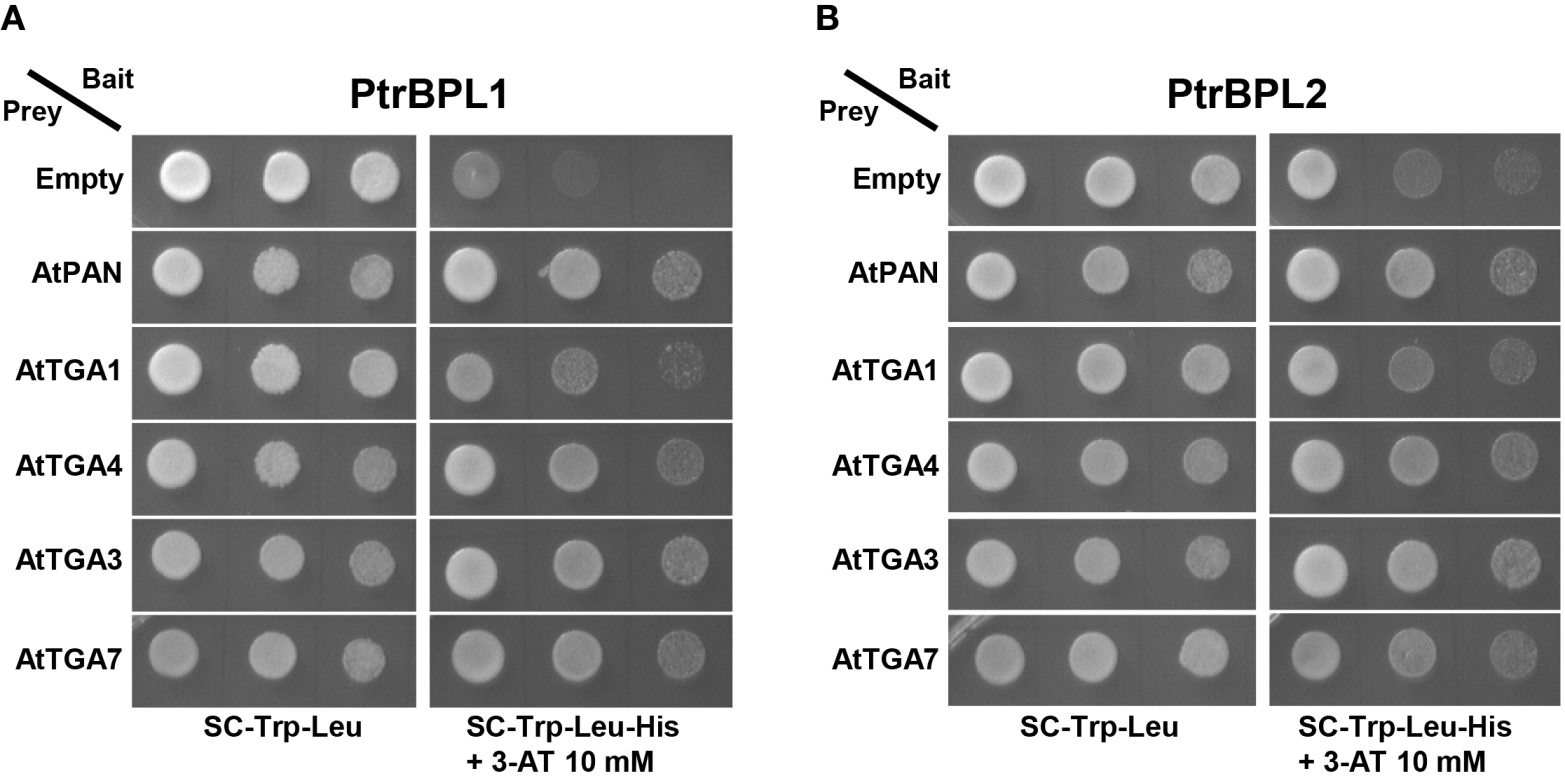
Pair-wise yeast two-hybrid assays showing **(A)** PtrBPL1 and **(B)** PtrBPL2 interaction with AtTGAs. PtrBPL1 and PtrBPL2 baits were fused to the DNA-binding domain of yeast GAL4. AtTGA1, AtTGA4, AtTGA3, and AtTGA7 preys were fused to the transcriptional activation domain of yeast GAL4. Plasmid constructs were co-transformed into yeast AH109 strain before serial dilutions (10^-1^, 10^-2^, 10^-3^) were plated onto SC/-Trp/-Leu medium with or without histidine + 10 mM 3-AT. Growth on SC/-Trp/-Leu/-His/+3-AT above background confirms a protein-protein interaction. Dilutions were spotted in replicate onto SC/-Trp/-Leu/-His/+3-AT medium. Photos on SC/-Trp/-Leu were taken three days after plating. For all assays, interaction with AtPAN/TGA8 was used as a positive control and an empty prey vector was used as a negative control.

BTB-ankyrin proteins perform a variety of function by utilizing TGA bZIP factors as interaction partners (Backer et al., 2019). For example, Arabidopsis BOP1 and BOP2 interact with clade I and clade III TGAs in yeast (Hepworth et al., 2005). Such complexes are implicated in the regulation of stem development and lignin deposition in plants (Wang et al., 2019; Zhang et al., 2019). Arabidopsis BOP1 and BOP2 also interact with PAN/TGA8 for patterning functions in the flower (Hepworth et al., 2005). A highly sensitive method for detecting protein-protein interactions, we used the yeast two-hybrid assay to assess PtrBPL interactions with AtTGA factors. PtrBPL1 and PtrBPL2 fused to the DNA-binding domain of the yeast transcription activator protein GAL4 were used as bait. AtTGA1, AtTGA4, AtTGA3, and AtTGA7 fused to the activation domain of GAL4 were used as prey. **Figure 5** shows that PtrBPL1 and PtrBPL2 interact with AtTGA1, AtTGA4, AtTGA3, and AtTGA7 providing evidence of conserved functional partners in Arabidopsis and poplar.

## Discussion

Trees display prominent radial growth in the stem in which secondary xylem and phloem tissues are produced by the vascular cambium. Current knowledge regarding the genetic regulation of cambium activity and secondary growth is still incomplete (Wang et al., 2021). Therefore, understanding gene families potentially involved in this process is desirable.

The BOP family of co-transcriptional regulators are conserved in dicots and monocots and contribute to numerous developmental processes. Studies in dicots such as tobacco (Wu et al., 2012), legumes (Frankowski et al., 2015; Couzigou et al., 2016; Magne et al., 2018), tomato (Xu et al., 2016; Izhaki et al., 2018) and monocots such as rice (Toriba et al., 2020), barley (Tavakol et al., 2015; Jost et al., 2016) and *Brachypodium* (Magne et al., 2020; Liu et al., 2022) reveal highly conserved functions, comparable to Arabidopsis, that influence organ patterning, inflorescence architecture, and abscission (Khan et al., 2014; Hepworth and Pautot, 2015). Taxon-specific roles in the maintenance of symbiotic nodule identity (Couzigou et al., 2012; Magne et al., 2018) and rhizome development (Toriba et al., 2020) have also been identified.

In flowering plants, BOPs are involved in boundary formation. These specific domains are important for the separation of meristematic regions from lateral organs (Aida and Tasaka, 2006; Žádníková and Simon, 2014). Compared to the shoot apical meristem, our knowledge of boundaries and their involvement in cambium meristems is limited. The conservation of BOPs across the plant kingdom (Khan et al., 2014) makes it plausible that orthologs in trees have roles in the vascular cambium and secondary growth.

The results shown here indicate that *PtrBPL1* and *PtrBPL2* play a role in secondary growth when overexpressed in Arabidopsis. *D35S:PtrBPL1* and *D35S:PtrBPL2* transgenic plants were characterized by dwarf stems with a thicker vascular ring and more developed phloem fibers whereas the increased activity of these genes in the hypocotyl interfered with xylem II production. Overexpression of either *PtrBPL1* or *PtrBPL2* resulted in phenotypes that were highly similar to those observed in Arabidopsis plants with overexpression of *AtBOP1* or *AtBOP2* (Khan et al., 2012b; Woerlen et al., 2017). Furthermore, the expression of *PtrBPL1* or *PtrBPL2* transgenes in a *bop1 bop2* mutant background was sufficient to rescue leaf and floral patterning defects. Abscission defects in the *bop1 bop2* mutant were also corrected. The *PtrBPL1p:GUS* and *PtrBPL2p:GUS* expression patterns in Arabidopsis plants were consistent with a role in boundary patterning and secondary growth. Finally, we identified a family of TGA bZIP factors in poplar with a clade structure similar that in Arabidopsis plants. Yeast two hybrid assays indicated that PtrBPL1 and PtrBPL2 can interact with AtTGAs known to mediate roles in plant development and lignin deposition. All of these results strongly suggest that BOP1/2-TGA modules characterized in Arabidopsis are conserved in *P. trichocarpa*. Therefore, it is feasible they contribute to stem development in trees.

In Arabidopsis plants, *BOP1* and *BOP2* genes are repressed by the class I KNOX homeodomain transcription factors SHOOT MERISTEMLESS (STM) and BREVIPEDICELLUS (BP) during meristem maintenance, stem development, and secondary xylem formation (Jun et al., 2010; Khan et al., 2012b; Liebsch et al., 2014; Khan et al., 2015; Woerlen et al., 2017). Consistent with these findings, *PtrBPL1* is significantly downregulated in the stem of hybrid poplar overexpressing the STM ortholog ARBORKNOX1 (ARK1) (Liu et al., 2015). Both ARK1 and ARK2 (ortholog of BP) are strongly expressed in the cambial zone (Groover et al., 2006; Du et al., 2009). In the ARK1 overexpressor, the boundary between the cambium and the secondary xylem was uneven and phloem fibers were reduced in number (Groover et al., 2006). In the ARK2 overexpressor, extra secondary phloem was produced at the expense of phloem fibers and secondary xylem leading to an overall decrease in the differentiation of lignified cell-types. Conversely, an *ark2* mutant generated by artificial miRNA silencing showed a premature differentiation of secondary xylem and phloem fibers, similar to the *bp* mutant in Arabidopsis (Du et al., 2009).

Studies in the tropical Cannabaceae tree *Parasponia andersonii* species complement these findings (Shen et al., 2021). CRISPR-Cas9 loss-of-function mutants in the *AtBOP1* ortholog *PanNODULE ROOT1 (PanNOOT1*) were altered in the development of xylem and phloem tissues without any obvious difference in cambium organization and size. Compared to the wild-type, the differentiation of secondary xylem and phloem was inhibited resulting in a reduced stem diameter. Transcriptomic data showed a reduction in the expression of lignin metabolism-related genes (Shen et al., 2021). In both Arabidopsis and cotton (*Gossypium hirsutum*) and the grass *Brachypodium distachyon* model, BOPs positively regulate the expression of lignin biosynthesis genes and lignin deposition in stems (Khan et al., 2012b; Zhang et al., 2019; Liu et al., 2022).

In Arabidopsis plants, BOP1 and BOP2 promote the expression of two homeobox genes to influence lignin deposition (Khan et al., 2012a; Khan et al., 2012b; Woerlen et al., 2017). Poplar plants contain homeobox genes that are highly similar to *ARABIDOPSIS HOMEOBOX GENE 1* (*ATH1*) (Potri.018G054700/Potri.006G230700) and *KNOTTED-LIKE FROM ARABIDOPSIS THALIANA 6* (*KNAT6*) (Potri.010G043500/Potri.008G188700) suggesting that downstream pathway components are conserved. The homologous genes in *P. andersonii* were significantly downregulated in *Pannoot1* stems showing a similar regulation as in Arabidopsis plants (Shen et al., 2021). The *PagKNAT2/6b* gene from a hybrid poplar clone (*P. alb*a X *P. glandulosa*) is highly expressed in xylem and phloem. Compared to controls plants, transgenic poplar clones overexpressing *PagKNAT2/6b* showed a reduction in xylem formation by negatively regulating the expression of NAC domain transcription factor genes including *PagXYLEM NAC DOMAIN 1* as a direct target (Zhao et al., 2020).

Spatial and temporal differences between *PtrBPL1p:GUS* and *PtrBPL2p:GUS* expression were observed. In Arabidopsis plants, the *PtrBPL1* promoter was active in xylem I whereas *PtrBPL2* the promoter was active at the boundary of the cambial zone and secondary phloem (**Figures 2A, B, D**). By contrast, a *PanNOOT1p:GUS* reporter was expressed in all three tissues during secondary growth (Shen et al., 2021). A similar pattern to *PanNOOT1* was detected in aspen (*P. tremula*) and birch (*Betula pendula*) using high-spatial-resolution gene expression mapping (Sundell et al., 2017; Alonso-Serra et al., 2019; Shen et al., 2021). By contrast, *AtBOP1p:GUS* and *AtBOP2p:GUS* expression is normally repressed by BP in the cambial zone and secondary xylem of the root and hypocotyl (Liebsch et al., 2014; Khan et al., 2015; Woerlen et al., 2017). Thus, divergent transcriptional regulation may account for phenotypic differences in secondary growth between species. Compared to *AtBOP1* and *AtBOP2*, the *PtrBPL1* and *PtrBPL2* promoters had a higher number of predicted binding sites for MYB-related and NAC transcription factors, which as a group contribute strongly to secondary growth in plants (Nakano et al., 2015).

Considering all of the above, BOP-TGA modules characterized in Arabidopsis are likely conserved in poplar. Information gained from this study forms a basis for further exploration of networks that regulate cambium development and secondary growth in trees.

## Data availability statement

The raw data supporting the conclusions of this article will be made available by the authors, without undue reservation.

## Author contributions

SH and EL designed the research. SL, BD, GA, JM, and EL performed the research. AB carried out bioinformatics analysis. SL, JM, and SH wrote the article. All authors contributed to the article and approved the submitted version.
