# Supplementary material for: Regulation of secondary growth by poplar BLADE-ON-PETIOLE genes in Arabidopsis
Source: Front Plant Sci. 2023 Nov 14;14:1244583. doi: 10.3389/fpls.2023.1244583 (PMC10682204; doi:10.3389/fpls.2023.1244583)
Supplement: Supplementary file 1 [file Presentation_1.pdf]

Supplementary Material

Regulation of secondary growth by poplar *BLADE-ON-PETIOLE* genes in Arabidopsis

Sibei Li, Bhaswati Devi, Gamalat Allam, Armaan Bhullar, Jhadeswar Murmu, Eryang Li, and Shelley R. Hepworth*

*Correspondence:
Shelley R. Hepworth
shelley.hepworth@carleton.ca

The following Supplementary Material is available for this article:

Figure S1. Alignment of Arabidopsis and poplar BTB-ankyrin proteins.

Figure S2. Phylogenetic tree showing relationships between Arabidopsis and poplar TGA proteins.

Figure S3. Expression patterns of *PtrBPL1p:GUS* and *PtrBPL2p:GUS* in different tissues.

Figure S4. Analysis of transcription factor binding sites in the promoter regions of *AtBOP1*, *AtBOP2*, *PtrBPL1*, and *PtrBPL2*.

Figure S5. Overexpression phenotype of *35S:BOP2* plants.

Table S1. List of primers.

Table S2. Summary of complementation phenotypes in Arabidopsis *bop1 bop2* mutants. transformed with *BOP1p:PtrBPL1* and *BOP1p:PtrBPL2* constructs.

Table S3. Summary of overexpression phenotypes in Arabidopsis wild-type plants transformed with *D35S:PtrBPL1* and *D35S:PtrBPL2* constructs.

```
AtNPR1  ------MDTTIDGFADSYEISSTSFVATDNTDSSIVYLAAEQVLTGPDVSALQLLSNSFESVFDSPDDFYSDAKLVL-SDGREVSFHRCVLSARSSFFKS 93
AtNPR2  -MMATTTTTTTARFSDSYEFSNTSGNSFFAAESSLDY--PTEFLTPPEVSALKLLSNCLESVFDSPETFYSDAKLVL-AGGREVSFHRCILSARIPVFKS 96
AtNPR3  -MATLTEPSSSLSFTSSHFSYGSIGSNHFSSSSA----------SNPEVVSLTKLSSNLEQLLSNSDCDYSDAEIIV--DGVPVGVHRCILAARSKFFQD 87
AtNPR4  MAATAIEPSSSISFTSSHLSNPSPVVTTYHSAA-----------------NLEELSSNLEQLLTNPDCDYTDAEIIIEEEANPVSVHRCVLAARSKFFLD 83
AtBOP1  ---------MSNTFEE----------------------------------SLKSMSLDYLNLLINGQA-FSDVTFSV--EGRLVHAHRCILAARSLFFRK 54
AtBOP2  ----------MSNLEE----------------------------------SLRSLSLDFLNLLINGQA-FSDVTFSV--EGRLVHAHRCILAARSLFFRK 53
PtrBPL1 -----------MTLED----------------------------------SLRTLSLDYLNLLINGQA-FSDVTFSV--EGRLVHAHRCILAARSLFFRK 52
PtrBPL2 -----------MTLED----------------------------------SLRSLSLDYLNLLINGQA-FSDVTFSV--EGRLVHAHRCILAARSLFFRK 52
                                                                       BTB/POZ

AtNPR1  ALAAAKKEKDSNNTAA---VKLEL---------KEIAKDYEVGFDSVVTVLAYVYSSRVRPPPKGV---SECADENCCHVACRPAVDFMLEVLYLAFIFK 178
AtNPR2  ALATVKEQKSSTT------VKLQL---------KEIARDYEVGFDSVVAVLAYVYSGRVRSPPKGA---SACVDDDCCHVACRSKVDFMVEVLYLSFVFQ 178
AtNPR3  LF---KKEKKISKTEK---PKYQL---------REMLPYGAVAHEAFLYFLSYIYTGRLKPFPLEV---STCVDPVCSHDCCRPAIDFVVQLMYASSVLQ 169
AtNPR4  LF---KKDKDSSEKKP----KYQM---------KDLLPYGNVGREAFLHFLSYIYTGRLKPFPIEV---STCVDSVCAHDSCKPAIDFAVELMYASFVFQ 164
AtBOP1  FFCESDPSQP--GAEP---ANQTGSGARAAAV-GGVIPVNSVGYEVFLLLLQFLYSGQVSIVPHKHEPRSNCGDRGCWHTHCTAAVDLSLDILAAARYFG 148
AtBOP2  FFCGTDSPQPVTGIDPTQHGSVPASPTRGSTAPAGIIPVNSVGYEVFLLLLQFLYSGQVSIVPQKHEPRPNCGERGCWHTHCSAAVDLALDTLAASRYFG 153
PtrBPL1 YFCGPDPPSGLDPSGS----RINLVGSPGSR--SNVIPVNSVGYEVFLLLLQFLYSGQVSIVPQKHEPRPNCGVRGCWHTHCTSAVDLALDTLAAARYFG 146
PtrBPL2 FFCGPDPPSGLDPSGS----RINTVGSPGSR--SNVIPVNSVGYEVFLLLLQFLYSGQVSIVPQKHEPRPNCGERGCWHTHCTSAVDLALDTLAAARYFG 146
                                                  BTB/POZ

AtNPR1  IPELITLYQRHLLDVVDKVVIEDTLVILKLANICGKACMKLLDRCKEIIVKSNVDMVSLEKSLPEELVKEIIDRRKELG--------------LE---VP 261
AtNPR2  IQELVTLYERQFLEIVDKVVVEDILVIFKLDTLCGTTYKKLLDRCIEIIVKSDIELVSLEKSLPQHIFKQIIDIREALC--------------LE---PP 261
AtNPR3  VPELVSSFQRRLCNFVEKTLVENVLPILMVAFNCKLT--QLLDQCIERVARSDLYRFCIEKEVPPEVAEKIKQLRLISP--------------QDEETSP 253
AtNPR4  IPDLVSSFQRKLRNYVEKSLVENVLPILLVAFHCDLT--QLLDQCIERVARSDLDRFCIEKELPLEVLEKIKQLRVKSV--------------N----IP 244
AtBOP1  VEQLALLTQKHLTSMVEKASIEDVMKVLIASRKQDMH--QLWTTCSYLIAKSGLPQEILAKHLPIELVAKIEELRLKSSMPLRSLMPHH----HDLTSTL 242
AtBOP2  VEQLALLTQKQLASMVEKASIEDVMKVLIASRKQDMH--QLWTTCSHLVAKSGLPPEILAKHLPIDVVTKIEELRLKSSIARRSLMPHNHH--HDLSVAQ 249
PtrBPL1 VEQLAMLTQKQLASMVDNASIDDVMKVLIASRKQDMH--QLWTTCSHLVAKSGLPPEVLAKHLPIDVVAKIEELRLKSSIARRSLMPHHHHHLHDLTAAA 244
PtrBPL2 VEQLAMLTQKQLANMVEKASIEDVMKVLIASRKQDMH--QLWTTCSHLVAKSGLPPEVLAKHLPIDVVAKIEELRLKSSIARRSLMPHHHHHLHDLTSAA 244
           BTB/POZ

AtNPR1  KVK----KHVSNVHKALDSDDIELVKLLLKEDHTNLDDACALHFAVAYCNVKTATDLLKLDLADVNHR-NPRGYTVLHVAAMRKEPQLILSLLEKGASAS 356
AtNPR2  KLE----RHVKNIYKALDSDDVELVKMLLLEGHTNLDEAYALHFAIAHCAVKTAYDLLELELADVNLR-NPRGYTVLHVAAMRKEPKLIISLIMKGANIL 356
AtNPR3  KISEKLLERIGKILKALDSDDVELVKLLLTESDITLDQANGLHYSVVYSDPKVVAEILALDMGDVNYR-NSRGYTVLHFAAMRREPSIIISLIDKGANAS 352
AtNPR4  EVEDKSIERTGKVLKALDSDDVELVKLLLTESDITLDQANGLHYAVAYSDPKVVTQVLDLDMADVNFR-NSRGYTVLHIAAMRREPTIIIPLIQKGANAS 343
AtBOP1  DLED---QKIRRMRRALDSSDVELVKLMVMGEGLNLDESLALIYAVENCSREVVKALLELGAADVNYPAGPTGKTALHIAAEMVSPDMVAVLLDHHADPN 339
AtBOP2  DLED---QKIRRMRRALDSSDVELVKLMVMGEGLNLDESLALHYAVESCSREVVKALLELGAADVNYPAGPAGKTPLHIAAEMVSPDMVAVLLDHHADPN 346
PtrBPL1 DLED---QKIRRMKRALDSSDVELVKLMVMGEGLNLNEALALHYAVENCSREVVKALLELGAADVNYPAGPAGKTPLHIAAEMVSPDMVSVLLDHHADPN 341
PtrBPL2 DLED---QKIRRMKRALDSSDVELVKLMVMGEGLNLDEALALHYAVENCSREVVKALLELGAANVNYQAGPAGKTPLHIAAEMVSPDMVAVLLDHHADPN 341
                              ANK1/2                                           ANK3/4

AtNPR1  EATLEGRTALMIAKQATMAVECNNIPEQCKHSLKGRLCVEILEQEDKREQI-PRDVPPSFAVAADELKMTLLDLENRVALAQRLFPTEAQAAMEIAEMKG 455
AtNPR2  DTTLDGRTALVIVKRLTKADDYKTSTEDGTPSLKGGLCIEVLEHEQKLEYLSPIEASLSLPVTPEELRMRLLYYENRVALARLLFPVETETVQGIAKLEE 456
AtNPR3  EFTSDGRSAVNILRRLTNPKDYHTKTAKGRESSKARLCIDILEREIRKNPMV-LDTPMCSISMPEDLQMRLLYLEKRVGLAQLFFPTEAKVAMDIGNVEG 451
AtNPR4  DFTFDGRSAVNICRRLTRPKDYHTKTSRKEPS-KYRLCIDILEREIRRNPLVSGDTPTCSHSMPEDLQMRLLYLEKRVGLAQLFFPAEANVAMDVANVEG 442
AtBOP1  VQTVDGITPLDILRTLTS------------------------------DFLFKGAIPGLTHIEPNKLRLCLELVQS--------------AALVISREEG 395
AtBOP2  VRTVGGITPLDILRTLTS------------------------------DFLFKGAVPGLTHIEPNKLRLCLELVQS--------------AAMVISREEG 402
PtrBPL1 VRTVDGITPLDILRTLTS------------------------------DFLFKGAVPGLVHIEPNKLRLCLELVQS--------------AASVLSREEG 397
PtrBPL2 VRTVDGVTPLDILRTLTS------------------------------DFLFKGAVPGLAHMEPNKLRLCLELVQS--------------AAMVLSREEG 397
                          ANK3/4

AtNPR1  TCEFIVT-SLEPDRLTGTKRTSPGVKIAPFRILEEHQSRLKALSKT---------------------------VELGKRFFPRCSAVLDQIMNC---EDL 524
AtNPR2  TCEFTAS-SLEPDHHIGEKRTSLDLNMAPFQIHEKHLSRLRALCKT---------------------------VELGKRYFKRC--SLDHFMDT---EDL 523
AtNPR3  TSEFTGL--SPPSSGLTGNLSQVDLNETPHMQTQRLLTRMVALMKT---------------------------VETGRRFFPYGSEVLDKYMAEYIDDDI 522
AtNPR4  TSECTGLLTPPPSNDTTENLGKVDLNETPYVQTKRMLTRMKALMKTGKSLRKCTFKFYSLTTRLTDSKPFNNAVETGRRYFPSCYEVLDKYMDQYMDEEI 542
AtBOP1  NNNS---------------------------------------------------------------------NDNNTMIYPRM-------------KDE 413
AtBOP2  NNSNNQN------------------------------------------------------------------NDNNTGIYPHM-------------NEE 423
PtrBPL1 SVN----------------------------------------------------------------------ATTSNPIYPPM-------------SDE 414
PtrBPL2 NV-----------------------------------------------------------------------NATSTLIYPSM-------------SDE 413

AtNPR1  TQLACGEDDTAEKRLQKKQRYMEIQ-----ETLKKAFSEDNL-ELGNSSLTDSTSSTSKSTGGKRSNRKLSHRRR---------------- 593
AtNPR2  NHLASVEEDTPEKRLQKKQRYMELQ-----ETLMKTFSEDKE-ECGKSS-------TPKPTSAVRSNRKLSHRRLKVDKRDFLKRPYGNGD 601
AtNPR3  LDDFHFEKGSTHERRLKRMRYRELK-----DDVQKAYSKDKESKIARSCLSAS---SSPSSSSIRDDLHNTT------------------- 586
AtNPR4  PDMSYPEKGTVKERRQKRMRYNELK-----NDVKKAYSKD---KVARSCLS-----SSSPASSLREALENPT------------------- 601
AtBOP1  HTSGSS--------LDSRLVYLNLG------ATNRDIGDDN--SNQREGMNLHHH-HHDPSTMYHHHHHHF-------------------- 467
AtBOP2  HNSGSS--GGSNNNLDSRLVYLNLGAGTGQMGPGRDQGDDH--NSQREGMSRHHHHHQDPSTMYHHHHQHHF------------------- 491
PtrBPL1 HNSSH------------------------------------------SSQRDHHAMSRHDPTMYRHHSHDF-------------------- 443
PtrBPL2 HNTSSSGSNLANLNLDSRLVYLNLGAAGSGQMGSRMDEED---DSNHNNQRDHHAMSRHDPTMYHHHSHDF-------------------- 481
```

Supplementary Figure 1. Alignment of Arabidopsis and poplar BTB-ankyrin proteins. Protein sequences were aligned using MUSCLE under default parameters in MEGA (www.megasoftware.net). The BOXSHADE alignment was prepared using Geneioius Prime 2022.1 (www.geneioius.com). Conserved motifs (BTB/POZ and ankyrin repeats) were identified using the SMART tool (www.heidelberg.de).

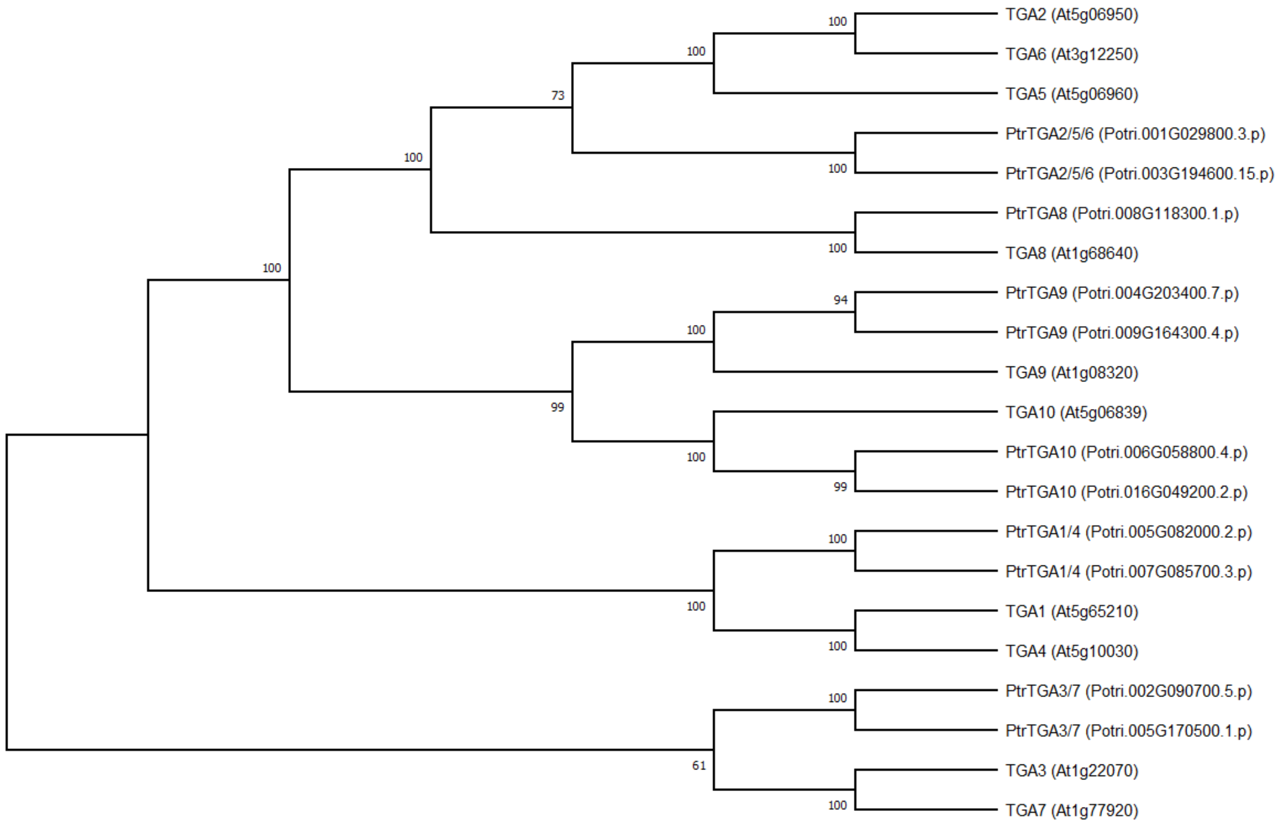

Supplementary Figure 2. Phylogenetic tree showing relationships between Arabidopsis and poplar TGA proteins. Protein sequences were aligned by MUSCLE under default parameters using MEGA (www.megasoftware.net). A maximum likelihood tree was constructed based on 100 bootstrap replicates using the Jones-Taylor-Thornton Model.

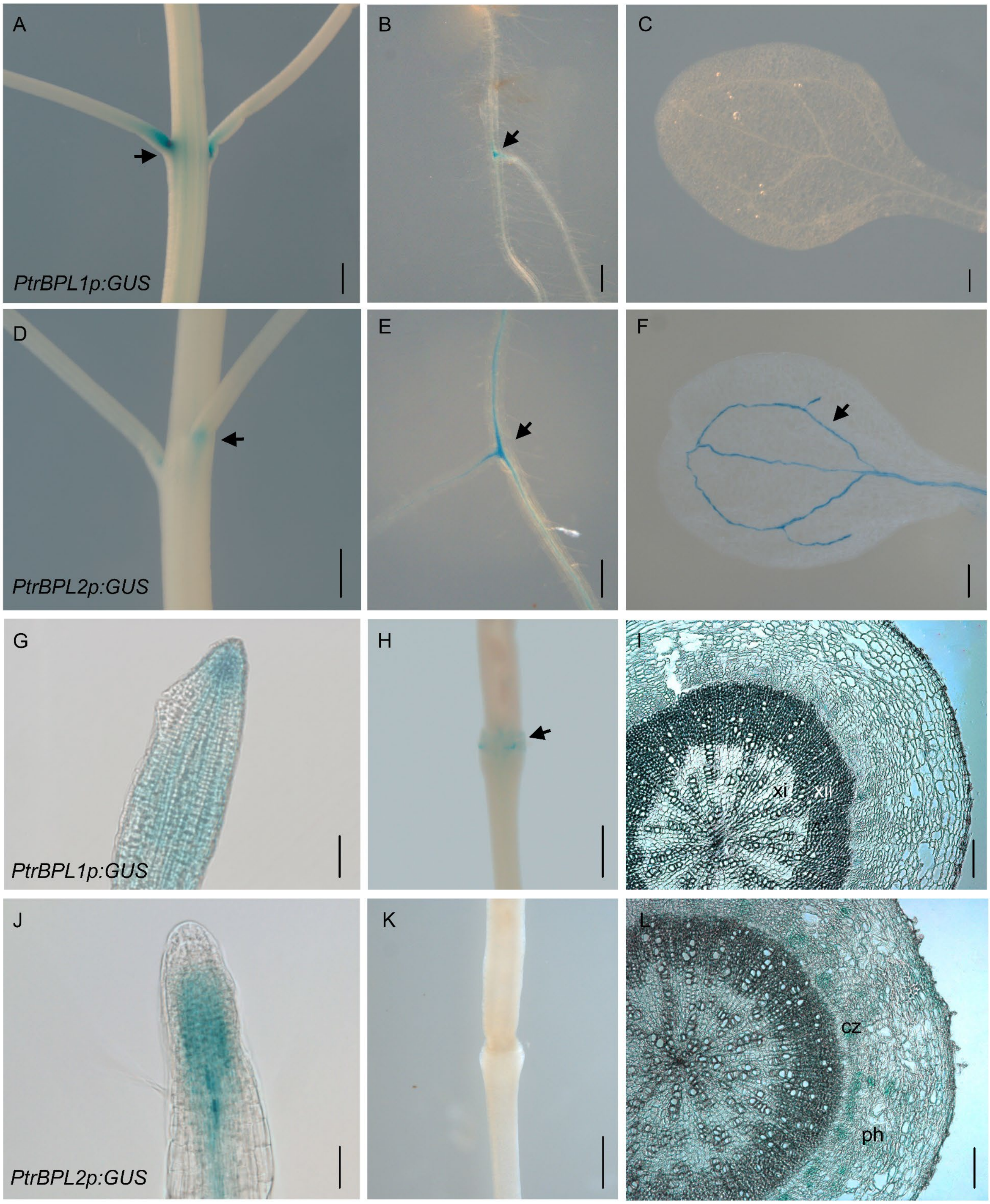

Supplementary Figure 3. Expression patterns of *PtrBPL1p:GUS* and *PtrBPL2p:GUS* in different tissues. Representative images are shown for constructs expressed heterologously in Arabidopsis plants. **(A)** and **(D)** showing the expression of both genes at the base of pedicels, see arrows. Scale bars, 500 µm. **(B)** and **(E)** showing the expression of both genes at the base of lateral roots, see arrows. Scale bars, 200 µm. **(C)** and **(F)** showing *PtrBPL2* expression in the vasculature of cotyledons, arrow. Scale bars, 200 µm. **(G)** and **(J)** showing the expression of both genes at the primary root tip. Scale bars, 50 µm. **(H)** and **(K)** showing *PtrBPL1* expression at the base of the silique in floral organ abscission zones, arrow. Scale bars, 500 µm. **(I)** and **(L)** showing expression in hypocotyl cross sections. *PtrBPL1* is expressed in the parenchyma and vessels of xylem I whereas *PtrBPL2* is express at the edge of the cambial zone and secondary xylem. Scale bars, 100 µm. xi, secondary xylem I, xii, secondary xylem II, ph, secondary phloem; cz, cambium zone.

A

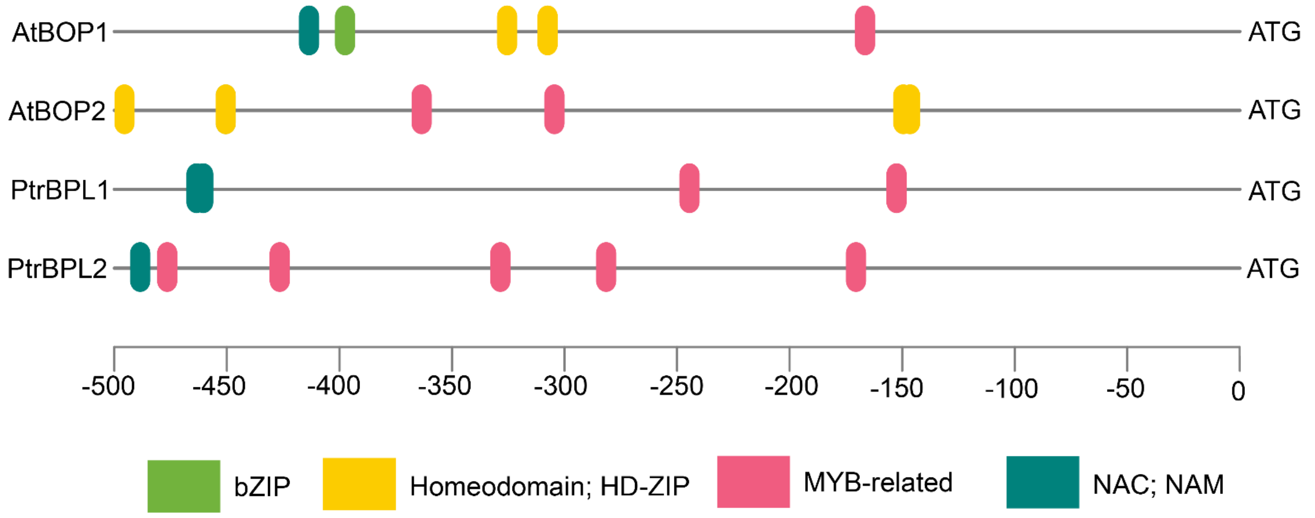

B

Family	oligoMer	p-value from binomial distribution
bZIP	TGATGA	1.33E-01
Homeodomain; HD-ZIP	ATTAAT	2.73E-02
	TAAATT	3.91E-01
	AATAAT	3.95E-01
MYB-related	ATATCT	1.64E-01
	AGATAT	1.64E-01
	AGAATC	8.38E-02
	TATCTT	2.04E-01
NAC; NAM	TTACTG	2.15E-02

Supplementary Figure 4. Analysis of transcription factor binding sites in the promoter regions of *AtBOP1*, *AtBOP2*, *PtrBPL1*, and *PtrBPL2*. Transcription Factor Binding Sites (TFBS) were identified using PlantPAN 3.0 (http://plantpan.itps.ncku.edu.tw/index.html). **(A)** Diagram showing selected TFBS location in the 500-bp promoter sequences upstream of the start codons of *AtBOP1*, *AtBOP2*, *PtrBPL1*, and *PtrBPL2* genes. The binding sites are shown by colored boxes prepared using TBtools v1.123. **(B)** Selected TFBS were matched to statistically enriched 6-mers in the four promoters identified using the TAIR motif analysis tool (https://www.arabidopsis.org/tools/bulk/motiffinder/index.jsp).

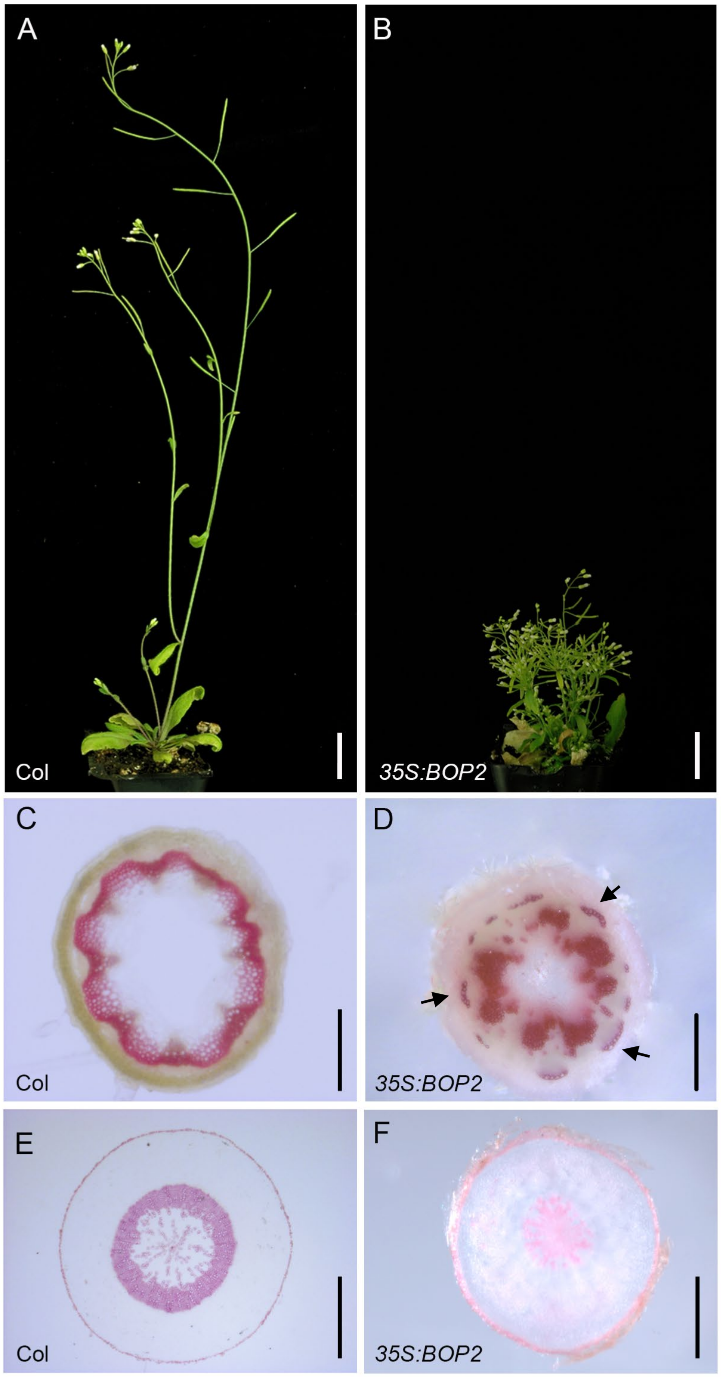

Supplementary Figure 5. Overexpression phenotype of *35S:BOP2* plants. (A) and **(B)** Representative flowering wild-type and *35S:BOP2* plants. **(A)** Wild-type plant, showing strong apical dominance and long internodes. **(B)** *35S:BOP2* plant, showing a bushy, dwarf stature. **(C)** and **(D)** Transverse sections from the base of fully elongated stems were stained phloroglucinol-HCl to reveal lignin (pink). Representative images are shown. **(C)** Wild-type stem, showing a continuous vascular ring. **(D)** *35S:BOP2* stem, showing a thicker, discontinuous vascular ring and lignified phloem fibers (arrows). **(E)** and **(F)** Transverse sections from the middle of the hypocotyl (1.5 mm below the rosette leaves) were stained phloroglucinol-HCl to reveal lignin (pink). Representative images are shown. **(E)** Wild-type hypocotyl, showing a thick ring of xylem II. **(F)** *35S:BOP2* hypocotyl, showing a lack of xylem II fibers and vessels. Scale bars: **(A-B)** 1.5 cm; **(C-D)** 0.25 mm; **(E-F)** 0.5 mm.

Supplementary Table 1. List of primers.

Primer name	Sequence (5'- 3')	Purpose
Pt6s04010UTR-F	ACGAGGCAACCCTAGCTAGTAAAC	PCR amplification of *PtrBPL1* cDNA
Pt6s04010UTR-R	CATTTTACTTGTCTAACTCTCCTGA	
Pt6s04190UTR-F	AGATAAATATACATATTTTTTGGAGTC	PCR amplification of *PtrBPL2* cDNA
Pt6s04190UTR-R	AACTTAGGTAGGCTTGGAACTAG	
Pt6s04010CDS-F	ATGACTCTTGAAGACTCTCTAAGAACTCTG	PCR amplification of *PtrBPL1* coding sequence
Pt6s04010CDS-R	CTAGAAGTCATGAGAGTGATGGC	
Pt6s04190CDS-F	ATGACTCTTGAAGACTCTCTAAGATCTCTA	PCR amplification of *PtrBPL2* coding sequence
Pt6s04190CDS-R	CTAGAAGTCATGAGAGTGATGGT	
PtrBPL-XbaI-F	CCATCTAGAATGACTCTTGAAGACTCTCTAAGA	Construction of *D35S:PtrBPL1* and *D35S:PtrBPL2* genes
PtrBPL-Sac1-R	CCAGAGCTCCTAGAAGTATGAGAGTGATGG	
PtrBPL1-pro-F10	CACCGCTAATATAGATTTAATGATGCCAATAAATG	Overlap PCR amplification of *PtrBPL1* promoter
PtrBPL1-pro-R1	GATATCCCTCAAAAATAAATTACCC	
BPL1-pro-F8	CCTAAGTATGTTTAGAGCTTTTACA	
BPL1-May14-R	ATGAACTAACCGACCCTCTACGCTGAA	
BPL1-pro-BamH1-F	CTAGAACTAGTGGATCCGCTAATATAGATTTAATGATGCCAATA AATG	Construction of *PtrBPL1p:GUS* reporter gene
BPL1-pro-Nco1-R	CAGGACGGACCATGGCATGAACTAACCGACCCTCTACGCTG	
BPL2-4kb-F(BamH1 site)	ATAGGATCCATGTGAGCATGCAGAGTTCAATAAAT	PCR amplification of *PtrBPL2* promoter
BPL2-pro-R (Pci1 site)	TCAACATGTGAGATAGAGATCTTAGAGAGTCTTCAAGAGTCAT	
BPL2-pro-BamH1-F	CTAGAACTAGTGGATCCATGTGAGCATGCAGAGTTCAATAAAT	Construction of *PtrBPL2p:GUS* reporter gene
BPL2-pro-Nco1-R	CAGGACGGACCATGGCAGATAGAGATCTTAGAGAGTCTTCAAGA GT	
PtBPL1 RT-F	ATGGGAGAAGGCCTCAATCTCA	RT-qPCR
PtBPL1 RT-R	GTCTCTCTGGCTGCTATGGC	
PtBPL2 RT-F	AGGCAGGGCCGGCAGGGAAAACT	RT-qPCR
PtBPL2 RT-R	CCTTGATCCCATTTGACCTG	

*Restriction enzyme sites are underlined.

Supplementary Table 2. Summary of complementation phenotypes in Arabidopsis *bop1 bop2* mutants transformed with *BOP1p:PtrBPL1* and *BOP1p:PtrBPL2* constructs.

BOP1p:PtrBPL1	**Leaf** (n=203 lines scored)				**Flower** (n=71 of 203 lines scored)			**Abscission** (n=160 of 203 lines scored)			
Complementation	+++	++	+	-	+++	++/+	-	+++	++	+	-
Number of transformants	42	68	5	88	46	6	19	92	31	20	72
BOP1p:PtrBPL2	**Leaf** (n=212 lines scored)				**Flower** (n=62 of 212 lines scored)			**Abscission** (n=182 of 212 lines scored)			
Complementation	+++	++	+	-	+++	++/+	-	+++	+++	+	-
Number of transformants	46	27	75	87	47	6	9	87	26	39	30

Arabidopsis *bop1 bop2* plants expressing *PtrBPL1* or *PtrBPL2* under a *BOP1* promoter were scored for complementation of leaf, flower, and abscission defects in the T1 generation. Degree of rescue was scored using the following key: complete or near complete (+++); moderate (++); weak (+); moderate to weak (++/+); no rescue (-). Plants with a complete rescue resembled the wild type. Plants with weak or no rescue resembled the *bop1 bop2* mutant with leafy petioles, asymmetric flowers with extra abaxial organs, and loss of floral organ abscission (described in Hepworth et al., 2005).

Supplementary Table 3. Summary of overexpression phenotypes in Arabidopsis wild-type plants transformed with *D35S:PtrBPL1* and *D35S:PtrBPL2* constructs.

**Construct**	**Number of transformants**	***bop1-6D* like (strong) phenotype**	***pny*-like (moderate) phenotype**
D35S:PtrBPL1	524	7	108
D35S:PtrBPL2	160	1	22

Arabidopsis wild-type plants expressing *PtrBPL1* or *PtrBPL2* under a double cauliflower mosaic virus promoter (D35S) were scored in the T1 generation. Two types of plant architectures were observed consistent with the known spectrum of *AtBOP* overexpression phenotypes: a *bop1-6D-like* phenotype: extremely short, bushy plants (<7 cm high) that are late-flowering with a wide leaf caused by strong constitutive *BOP* expression; and a *pny*-like phenotype: short, bushy plants (<12 cm high) with clusters of fruits caused by moderate *BOP* misexpression in the stem (described in Khan et al., 2015).
